# Effects of Noonan Syndrome-Germline Mutations on Mitochondria and Energy Metabolism

**DOI:** 10.3390/cells11193099

**Published:** 2022-10-01

**Authors:** Donald Bajia, Emanuela Bottani, Katarzyna Derwich

**Affiliations:** 1 Department of Pediatric Oncology, Hematology and Transplantology, Poznan University of Medical Sciences, Ul. Fredry 10, 61701 Poznan, Poland; 2 Department of Diagnostics and Public Health, Section of Pharmacology, University of Verona, Piazzale L. A. Scuro 10, 37134 Verona, Italy

**Keywords:** RASopathies, mitochondria, energy metabolism, OXPHOS

## Abstract

Noonan syndrome (NS) and related Noonan syndrome with multiple lentigines (NSML) contribute to the pathogenesis of human diseases in the RASopathy family. This family of genetic disorders constitute one of the largest groups of developmental disorders with variable penetrance and severity, associated with distinctive congenital disabilities, including facial features, cardiopathies, growth and skeletal abnormalities, developmental delay/mental retardation, and tumor predisposition. NS was first clinically described decades ago, and several genes have since been identified, providing a molecular foundation to understand their physiopathology and identify targets for therapeutic strategies. These genes encode proteins that participate in, or regulate, RAS/MAPK signalling. The RAS pathway regulates cellular metabolism by controlling mitochondrial homeostasis, dynamics, and energy production; however, little is known about the role of mitochondrial metabolism in NS and NSML. This manuscript comprehensively reviews the most frequently mutated genes responsible for NS and NSML, covering their role in the current knowledge of cellular signalling pathways, and focuses on the pathophysiological outcomes on mitochondria and energy metabolism.

## 1. Introduction

RASopathies are one of the most prominent groups of developmental disorders in humans with mutations in genes encoding proteins involved in the RAS/MAPK cascade. Over 20 genes have been associated with RASopathies so far [1]. Since the RAS pathway regulates cell cycle, cell growth, proliferation, differentiation, and metabolism, and it plays a crucial role in developing and maintaining homeostasis in different tissues, it is not surprising that its genetic dysregulations lead to severe clinical complications. The RASopathies include Noonan Syndrome [NS; Mendelian Inheritance in Men (MIM) #163950] and Noonan Syndrome with Multiple Lentigines [NSML, also termed LEOPARD syndrome; MIM #151100], and other related syndromes, which share overlapping clinical phenotypes spanning from developmental delay and reduced cognitive skills to heart defects and early-onset cancer [2]. Pre-diagnosis is based on recognising clinical phenotypes and is confirmed through molecular genetic testing. An accurate diagnosis improves the management of patients’ symptoms and aids in designing clinical trials to develop potential treatments for the disease [3]. Mitochondria are the major source of adenosine triphosphate (ATP), synthesized by the mitochondrial respiratory chain through the process of oxidative phosphorylation (OXPHOS). ATP is the primary energy substrate required for all active processes within the cells, and ATP deficiency leads to cellular dysfunction and, ultimately, cell death. Though not all encoded proteins of NS/NSML genes have been reported directly localized to the mitochondria, research efforts suggest they might still play a role in mitochondrial function since they are important regulators of RAS signalling [1,4,5,6,7]. RAS proteins and associated pathways have been extensively studied for their role in disease development, revealing their regulation and significance in cellular proliferation, differentiation, and survival [8]. Considering studies have tried to link RAS signalling to energy metabolism and human diseases [1]. From that perspective, we focus on understanding how NS germline mutations affect mitochondrial metabolism by looking at the most commonly mutated genes responsible for the pathogenesis of associated human diseases. Noteworthy that NS/NSML are considered a model disorder of the RASopathy family given its clinical peculiarity and challenges among the other RASopathies [9,10].

This review describes the pathophysiological impact of NS and NSML-related germline mutations on cell metabolism. We revise the current knowledge of RAS/MAPK pathway dysregulation and its pathophysiological consequences on mitochondria and energy metabolism. Finally, we summarise the overall remarks, including therapeutic perspectives and future directions.

## 2. The RAS/MAPK Pathway

The RAS-MAPK pathway plays a profound role in developmental, endocrinal and metabolic processes, which comprises the cascade of downstream signals involving a coordinated action of proteins stimulated by extracellular factors. This signalling cascade incorporates complex and multiple levels of regulation, including transcriptional, post-translational, protein-lipid interactions and crosstalk with other signalling pathways. Here, we focus on the main elements of NS and NSML involved with this pathway while referring our readers to detailed information [8,11] for full knowledge of other components of the pathway.

The activation of the signal cascade starts with the binding of ligands (mitogens) such as growth factor (GF) and epidermal growth factors (EGFS) to cell surface receptors allowing a small Ras protein (GTPase) to swap GDP molecule for a GTP molecule, flipping the “on/off switch” of the pathway. The ligand-binding causes autophosphorylation of tyrosine residues, resulting in the formation of a binding site for GRB2 adaptor protein and recruitment of the guanine nucleotide exchange factor SOS. Activated SOS promotes the removal of GDP from a member of the Ras subfamily (particularly H and K Ras), allowing RAS binding to GTP to become active. Apart from SOS, another key player, the tyrosine phosphatase SHP2, can activate Ras by dephosphorylating a series of inhibitory kinase proteins on docking proteins (src regulatory proteins) or RAS itself [12,13]. Activated Ras then activates RAF, the first protein kinase of the so-called MAPK pathway [14]. Dephosphorylating PP1C (protein phosphatase 1 catalytic subunit gamma), an inhibitory kinase activated by SHOC protein, is required for full RAF activation. RAF kinase then phosphorylates and activates MAPK/ERK Kinase (MEK1 or MEK2). ERK then phosphorylates and activates cytoplasmic and nuclear components such as translation regulators and transcription factors, initiating a sequence of cellular responses.

## 3. Noonan Syndrome (NS) and Noonan Syndrome with Multiple Lentigines (NSML)

NS is a relatively common autosomal-dominant genetic disorder with an incidence of 1 per 2000–2500 live births that was first described by pediatric cardiologist Jacqueline Noonan 53 years ago [15]. NS is characterised by distinctive phenotypic traits such as abnormal facial features (i.e., hypertelorism, ptosis, down slanting palpebral fissures, low-set posteriorly rotated ears, and short/webbed neck), cardiopathies, growth retardation including learning disabilities, cryptorchidism in males, predisposition to myeloproliferative disorders, and endocrine/metabolic imbalance. Due to the high mutation rates and heterogeneity in NS from other RASopathies, diagnosis among NS patients has been challenging [9,10]. Distinctive facial features are prominent from birth until middle childhood and decrease in adulthood [16]. Germline mutations described in NS and NSML patients, their locations, and mutational rate are presented in Table 1, with a high mutation prevalence observed on the PTPN11 gene. Variations in mutated genes of NS consequently lead to overlapping phenotypes with characteristic features resembling that of other RASopathies. As observed in NSML, the mutations PTPN11, BRAF and RAF1 account for approximately 95% of mutations in NS; thus, the phenotypic resemblance though NSML has its own distinctive features characterised by multiple lentigines dispersed throughout the body, café-au-lait spots, and a higher prevalence of electrocardiographic conduction abnormalities, obstructive cardiomyopathy and sensorineural hearing deficits [17,18].

## 4. Energy Metabolism and the Oxidative Phosphorylation (OXPHOS) Machinery

Metabolism is generally defined as the collection of biochemical reactions that either produce (catabolism) or consume (anabolism) energy. Understanding the physiological mechanisms that regulate cell metabolism, and the pathophysiological consequences of its dysregulation have been among the most fruitful pursuits in disease-oriented research [36]. The amount of ATP, AMP, ADP, and phosphocreatine/creatine in a cell, as well as the availability of glucose, amino acids, or fatty acids determines the state of intracellular energy [37].

Nutrients required by cells to generate energy in the form of nucleotides (ATP and GTP) [38] are metabolised and shuttled into the tricarboxylic acid (TCA) cycle, where electrons are stored in the reducing equivalents NADH and FADH_2_ via oxidative processes [38]. The electron carriers NADH and FADH_2_ move electrons into the electron transport chain (ETC) of the inner mitochondrial membrane which uses electron flow to pump protons into the intermembranous space (IMS) [39]. The OXPHOS machinery consists of an ETC and the F0-F1 ATP synthase, also known as complex V. The ETC is a group of multiprotein-complexes that include NADH dehydrogenase (complex I), succinate dehydrogenase (complex II), coenzyme Q: cytochrome c–oxidoreductase (complex III), and cytochrome c oxidase (COX, complex IV) plus two electron-shuttle molecules, Coenzyme Q and Cytochrome C. Electron transport is coupled to proton pumping via complexes I, III and IV, resulting in the generation of a mitochondrial membrane potential (ΔΨm) across the inner mitochondrial membrane. This membrane potential is utilized by ATP synthase to synthesize ATP from ADP and phosphate via the counterflow of protons from the mitochondrial IMS to the matrix. Though OXPHOS accounts for most of the energy produced in the cell, it is accompanied by physiological production of reactive oxygen species [40].

Cellular metabolic changes are likely due to the numerous cellular processes and signalling pathways impacted by RAS. Dard et al. described the involvement of RAF-MEK-ERK pathway in activating transcription factors which induce several genes responsible for cell cycle progression and migration, cell survival, cell growth, endocytosis and calcium signalling, thus contributing profoundly to the cells’ mitochondrial dynamics and homoestastis [1]. A study by Feramisco et al., showed that RAS is sufficient to stimulate the proliferation of dormant cells [41]. Thus, the RAS proteins that regulate cell activity also regulate energy metabolism via the RAF-MEK-ERK and PI3K-AKT-mTOR pathways [42]. One of the mechanisms for modulating cellular energy and cell growth involves S6 kinases 1 and 2 (S6Ks) and the AMP-activated protein kinases (AMPKs) signal transduction. Phosphorylation of AMPK suppresses the sensitivity of cell growth to nutrient availability and mTOR/S6K, thus activating catabolic processes to release ATP. Likewise, inhibition of AMPK turns on the anabolic pathways utilising ATP [43].

Many diseases such as mitochondrial diseases [44,45], metabolic syndrome [46], neurodegenerative diseases [47,48,49,50] and cancer [48,51,52] arise when the mitochondria are perturbed by genetic or environmental toxin-mediated cues causing defects in the structural composition of the mitochondrial membrane, defects in ETC protein complexes, defects of mitochondrial protein import or defects in mitochondrial motility [44,45,49,50,53] among others. However, the impacts of mutations in genes involved in the RAS signalling pathway on mitochondria and energy metabolism have emerged only recently. Thus, in the next section, we discuss the pathophysiological consequences of NS mutations in signalling pathways on mitochondria metabolism.

## 5. Functional and Pathophysiological Effects of NS and NSML Mutations on Mitochondria and Energy Metabolism

### 5.1. PTPN11/SHP2 

*Ptpn11*, the gene found most frequently mutated in NS and NSML [19,20,34], encodes for SHP2 (Src-homology 2 domain-containing phosphatase 2), a non-receptor tyrosine phosphatase (PTP). SHP2 structure comprises two N-terminal Src homology 2 (SH2) domains and a central tyrosine phosphatase domain. SHP-2 is found in various tissues and cell types, where it targets FGF (fibroblast growth factor), cytokines such as IL-3, GM-CSF, and EPO, as well as insulin and interferon receptors. The central tyrosine phosphatase domain docks several signalling intermediate proteins in the cytoplasm, including Grb2, FRS-2, Jak2, the p85 subunit of PI3 kinase, IRS-1, and Gab1 and 2 [54]. SHP2 has also enzymatic activity on specific membrane proteins such as leukaemia inhibitory factor (LIF), in which it binds and reduces the level of this LIF, thereby mediating the JAK/STAT3 pathway [55]. SHP2 bound to LIF receptor (LIFR) dephosphorylates LIF. The dephohosphorylation of LIF inactivates JAK/STAT3 signalling resulting in the inhibition of cell proliferation in mouse embryonic stem cells [56,57]. The C-terminus of SHP2 contains two regulatory phosphorylation sites required for downstream MAPK activation via fibroblast growth factor and platelet-derived growth factor, but not epidermal growth factor signalling [58]. Shp2 is a unique player in signalling pathways such as Janus-tyrosine kinase 2 (Jak2)/signal transducer and activator of transcription (STAT3) and mitogen-activated protein kinase (MAPK) [59], thus suggesting its role in cell development, growth, and survival. According to Saxton and colleagues, homozygous mice with dysfunctional SHP2 lacking the N-terminal SH2 domain-required for phosphotyrosine recognition-die before E.11.5 of gestation [60]. Likewise, *Ptpn11*-null mice die much earlier during the peri-implantation period [61]. Notably, mutated *Ptpn11* is not the only cause of NS or NSML but also other haematological malignancies such as leukaemia [62,63,64,65]. Mostly, NS mutations lead to constitutive active SHP2, and in particular, the D61G substitution (PTPN11^D61G^) results in a 10-fold increase of basal SHP2 activity, while the leukaemia-causing D61Y change (PTPN11^D61Y^) induces 20-fold SHP2 basal activity [66]. Interestingly, SHP2 was found located in the intercristae/intermembrane space (IMS) of mitochondria from rat brain [5,6,67,68].

A recent study in vivo illustrated the role of SHP2 as a critical regulator of MAPK and PI3K signalling pathways in development and metabolism homeostasis. Male mice with a gain-of-function (GOF) T468M heterozygous mutation (Ptpn11^T468M/+^) causing a complex NSML phenotype showed reduced adipocytes in their epididymal adipose tissue resulting from increased metabolic activity. In particular, an enhanced lipolytic effect was due to the overexpression of HSL and ATGL, key lipases in the adipose tissue [69], which was paralleled by a higher rate of glycerol release from the adipose tissue, thus enhancing lipolysis. It should be noted that enhanced lipolysis contributes to defects in adipose tissue maintenance. The consequences of reduced lipogenesis on whole-body metabolism were assessed in *Ptpn11*^T468M/+^ and *Ptpn11*^Y279C/+^ mice by oral glucose tolerance test (OGTT). Increased glucose tolerance was observed due to enhanced insulin sensitivity, thus suggesting NSML mice display a lean phenotype [69]. This phenotype corresponded with defective adipogenesis, better insulin signalling, and enhanced energy expenditure and was reverted with MEK inhibitors and Rapamycin, suggesting activating *Ptptn11* mutation alters energy balance through hormonal regulation [69]. However, in another study, dominant-active mutant SHP2^D61A^ transgenic mice showed resistance to high-fat diet (HFD)-induced obesity, followed by improved insulin resistance and glucose homeostasis. This phenotype was accompanied by an enhanced energy expenditure and reduced food intake was observed only in females due to the synergistic action of leptin and estrogen [70]. This synergistic effect was observed by enhanced Erk phosphorylation when combined hormones were injected into the SHP2^D61A^ mouse. Since SHP2 is essential for controlling specific physiological processes and metabolism, genetic ablation in the central nervous system results in gross obesity associated with leptin resistance and decreased p-Erk [71,72]. Despite the positive role of an active SHP2 in managing obesity by activating leptin receptors (LepRb) in the hypothalamus, threatening consequences of active mutant Shp2 exist, such as the development of tumors [73].

Kleefstra et al. conducted a study on 18 patients diagnosed with RAS/MAPK defects, with most of them exhibiting clinical manifestations of NS and NSML. Molecular analysis identified heterozygous de novo pathogenic mutations in BRAF (p.Thr241Pro), *PTPN11* (p.Thr42Ala, p.Tyr279Ser), KRAS, and HRAS. Metabolic screening of five patients revealed lactic acidemia in serum, 3-methylglutaconic aciduria-known markers for mitochondrial dysfunction [74] and increased urinary ethylmalonic acid excretion. All patients showed a mitochondrial disease criterion (MDC) score greater than 6, indicating mitochondrial dysfunction. Biochemical analysis of muscular mitochondrial function in four patients indicated an altered OXPHOS, i.e., decrease in complex I and multiple complex deficiencies (complexes II, III) and a significant drop in ATP production. [75,76].

Xu et al., demonstrated that the activating heterozygous *Ptpn11*^D61G/+^ mutation in mice increased ROS levels with a decreased buffer capacity of H_2_O_2_ in fresh bone marrow cells. The higher levels of ROS did not originate from hematopoietic stem cells (HSCs) but rather from Myeloid progenitors, including common myeloid progenitors (CMPs), granulocyte-macrophage progenitors (GMPs), and megakaryocyte erythroid progenitors (MEPs). This was confirmed also in *Ptpn11*^D61G/+^/*Ptpn11*^E76K/+^ myeloid progenitors. Further, bone marrow *Ptpn11*^D61G/+^ cells cultured with IL-3 showed a tremendous increase in ROS compared to *Ptpn11*^+/+^ control cells [77]. Interestingly, several findings of *Ptpn11* GOF mutations associated with Juvenile myelomonocytic leukaemia (JMML), a common clinical feature of NS and NSML are characterised by cytokine hypersensitivity in myeloid progenitors [62,64,78]. Thus, cytokine signalling could be responsible for controlling ROS-induced cellular responses. Since SHP2 also localises in the mitochondria [5,6], Xu et al., hypothesised that there might be substrates or proteins interacting with SHP2 to provide a mechanistic insight of GOF *Ptpn11* mutation on mitochondria metabolism; they identified an135 kDa tyrosine-phosphorylated protein (p135) trapped by Shp2 D425A in murine pro B-cells (BAF3), suggesting that the protein might be responsible for the pathogenicity of GOF mutant *Ptpn11* on mitochondrial metabolism and related malignancies seen in NS patients [77]. Arachiche and coworkers identified SHP2 specifically localised in brain mitochondria, and showed that ATP addition enhanced SHP2 in brain mitochondria. Further enhancement was observed in the presence of the tyrosine phosphatase inhibitor orthovanadate, suggesting that SHP2 may play a vital role in brain mitochondrial respiration through tyrosine phosphorylation of complexes mediated by tyrosine-protein kinase Src autoactivation [6].

Studies of constitutively active SHP2 (D61G) on mouse embryonic fibroblasts (MEF) showed deregulation of cytochrome c oxidase subunits and activity associated with a decrease in mitochondrial membrane potential (MMP) [7]. Therefore, SHP2 (D61G) might affect proton pumping across the complexes of the ETC due to the down-regulated cytochrome c oxidase subunits. Kadenbach proposed that dephosphorylation of COX causes a “slide” in proton pumping activity, i.e., a decrease in H+/e− stoichiometry, thus leading to decreased mitochondrial membrane potential and ATP production [79]. Lee and colleagues suggested that decreasing MMP and ATP production is associated with the D61G cells’ high energy demands since an increased growth rate was observed in these cells compared to the controls [7]. In summary, alterations of SHP2 could induce changes in MMP critical for mitochondrial ATP and ROS production. Lee et al. speculated that these changes in MMP are caused by the exchange of a vast number of ions, elements, and molecules with the cytoplasm due to prolonged opening of mitochondria membrane pores [7].

Ptpn11 is a critical gene for mitochondrial function [80]. The expression level of SHP2 on mitochondria was regulated by a growth arrest-specific gene 6 (*Gas6*) [80]. This was observed through a qPCR evaluation to identify transcripts associated with increased *Ptpn11* in oocytes culture of female mice subjected to *Gas6* suppression. Previously, the authors identified that oocytes in which *Gas6* was deleted showed insufficient oocyte maturation due to excessive mitochondrial activation. Intriguingly, mitochondrial over-activation was accompanied by the inhibition of mitophagy leading to the cumulation of dysfunctional mitochondria [81]. Thus, suggesting increased levels of *Ptpn11* due to the downregulation of *Gas6* might be associated with changes in mitochondrial respiratory function [80]. Though, unfortunately, the authors failed to assess the mitochondrial respiratory function i.e., MMP, ETC complexes and ATP production. 

In a study by Zheng et al., inducible *Ptpn11*^E76K^ Knock-in MEFs showed elevated levels of intracellular ROS, and of mitochondrial respiration through an unknown mechanism [82]. These data were confirmed in mitochondria from liver-specific *Ptpn11*^E76K/+^ knock-in mice which had higher respiration but decreased coupling efficiency [82]. Additionally, *Ptpn11* KO MEF had reduced ATP and ROS levels [82]. Together, these findings suggest that this particular GOF SHP2 phenotype enhances mitochondrial oxygen consumption because of reduced coupling efficiency, which is a general indicator of mitochondrial dysfunction [83] while its absence reduces mitochondrial metabolism.

SHP2 also regulates mitochondrial Cyt C release and apoptosis. In rat cardiac myocytes, 48 h of serum deprivation caused Cyt C release, and this effect was greatly enhanced by concomitant administration of NSC87877, an inhibitor of SHP2. These findings suggested that inhibiting SHP2 might promote apoptosis by favouring the release of Cyt C from the mitochondria [84,85].

**Figure 1:**
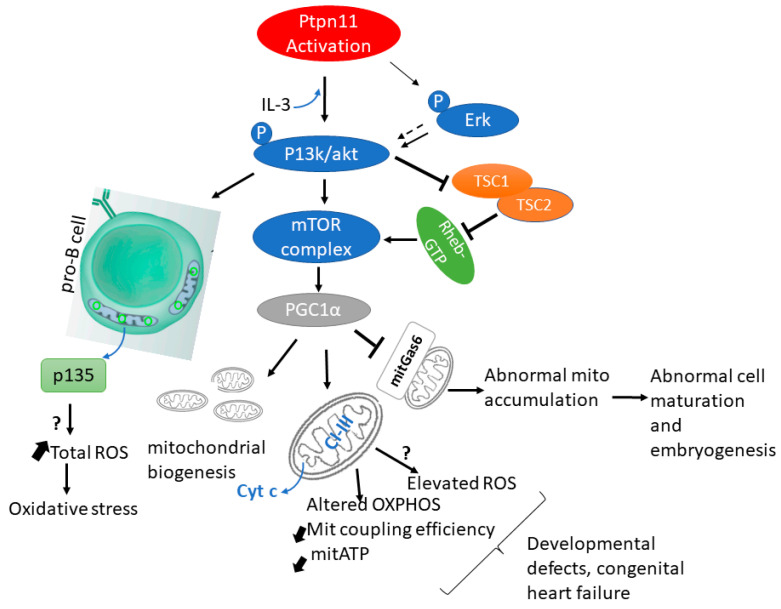
**Activating *Ptpn11* mutation through Erk/PI3k/Akt/mTOR signalling affects mitochondrial function.** The presence of IL-3 (upward-blue arrow), active *ptpn11* activates p13k/Akt signalling which inturn triggers the release of a mitochondria protein, p135 through an unknown mechanism(?). p135 release causes an increase (up-slanted arrows) in total ROS responsible for oxidative stress in tissues. Activated PI3K/Akt activates mTOR. Hypeactivated mTOR blocks the mitochondrial Growth arrest protein (mitGas6) leading to abnormal cell maturation. Via hyperactivated mTOR, Ptpn11 alters mitochondria respiration by decreasing (down-slanted arrows) CI-III of the ETC and coupling efficiency, releasing cyt c to the cytoplasm and reducing mitochondrial ATP.

The metabolic effects of hepatic SHP2 deletion in mice fed with a high-fat diet (HFD) have been studied to investigate further its role in lipid metabolism and energy balance [85]. Liver-specific SHP2 knockout (LSHKO) mice fed with HFD gained significantly less body weight than controls, suggesting hepatic SHP2 deficiency affects systemic energy balance. Notably, the energy expenditure was significantly higher in LSHKO mice than in controls. Thus, these findings suggest hepatic SHP2 knockout increased whole-body energy expenditure [85]. Taken together, these data indicate that GOF *Ptnp11* mutations affect mitochondria function and metabolic homeostasis via phosphorylation and hyperactivation of Erk, and P13K/Akt signalling resulting in mitochondrial complex deficiencies, decrease coupling efficiency, Cyt C release, mitochondrial hyperactivation and abnormal mitochondrial accumulation, excessive ROS production, as well as altered lipogenesis and energy balance. These defects together result in the phenotypes observed in NS models and patients, (Figure 1) and connect the mechanistic insights and outcomes of *Ptpn11* mutation on mitochondria metabolism.

STAT3, a known substrate of SHP2 phosphatase [86,87], plays a vital role in the mitochondria [88]. The phosphorylation levels of STAT3 at Tyr705 and Ser727 were significantly reduced in *Ptpn11*^E76K/+^ cells and isolated mitochondria [84]. STAT3 localised in the mitochondria of cultured cells and primary tissues such as liver and heart. Furthermore, complex I and II-driven respirations were reduced in STAT3^-/-^ mitochondria, confirming defects in mitochondrial respiration in the absence of STAT3 [88]. Furthermore, STAT3 deficiency in splenocytes increased mitochondrial oxygen consumption and ROS levels not related to ATP synthesis [89], similar to the mitochondrial phenotypes in *Ptpn11*^E76K/+^ cells described above [82]. 

Nagata and coworkers examined STAT3 phosphorylation to determine the molecular basis for increased energy expenditure in LSHKO mice. SHP2 has been linked to the regulation of STAT3 phosphorylation on Tyr705 in mouse heart [90] and liver [91]. Notably, this phosphorylation activates STAT3, and phosphorylation at Ser727 is required for maximal STAT3 transcriptional activity [88,92] for its mitochondrial actions and regulation of OXPHOS [88,93]. STAT3 (Tyr705) phosphorylation was increased in LSHKO liver lysates and isolated mitochondrial fractions compared to control mice. Together, findings show that total and mitochondrial STAT3 phosphorylation increased in HFD-LSHKO mice, implying that SHP2 deficiency may contribute to increased energy expenditure [85].

Finally, to check whether increased STAT3 (Ser727) phosphorylation may affect mitochondrial respiration, the oxygen consumption rate (OCR) of LSHKO and control mitochondria was measured. ADP-stimulated respiration rate in LSHKO mitochondria was significantly higher than in control mice. Furthermore, LSHKO mice had a significantly higher complex III-linked reactive oxygen species production than control mice. These findings show that hepatic SHP2 modulates mitochondrial function, including respiration and electron transport, in the liver of HFD-fed mice, which may contribute, at least in part, to the increased energy expenditure in LSHKO mice [85]. However, additional studies are needed to understand better the SHP2 regulation of mitochondrial function. 

**Figure 2:**
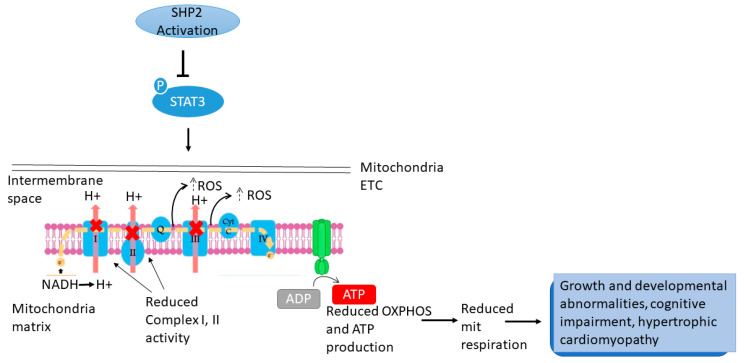
**Activating SHP2 and STAT3 signalling in mitochondrial metabolism.** Mutated SHP2 blocks STAT3 phosphorylation which inturn initiates a cascade of actions in the mitochondrial ETC. Reduced complex I, II activity results in deficient OXPHOS and ATP generation and is accompanied by increased ROS (upward dotted arrows), thus decreasing mitochondria respiration resulting in phenotypes observed in NS/NNSML.

Together, these data indicated that, the inhibition (dephosphorylation) of STAT3 accounts for at least part of the changes in mitochondria function associated with SHP2 mutations. These changes include reduction in CI and II activity accompanied by decreased OXPHOS and ATP production, and increased ROS production in cellular models (Figure 2).

### 5.2. KRAS 

GOF KRAS mutations (*KRAS*^G12V^, *KRAS*^Y71H^) cause Erk activation and cell proliferation [94]. Heart and craniofacial abnormalities were observed in KRAS-targeted morpholino knockdown zebrafish embryos, while the expression of mutant KRAS caused heart maldevelopment [95]. A recent study on an NS patient’s DNA with a heterozygous de novo mutation c.211T>G, p.Tyr71Asp identified in exon 2 of *KRAS* described clinical features of intellectual impairment and nerve root hypertrophy, though the onset mechanism remains unknown [21]. However, only a few studies have investigated the consequences of *KRAS* mutations on mitochondria and cellular metabolism to this extent.

A tetracycline-inducible human embryonic kidney (HEK293) cell model was used to demonstrate that activation of mutated *K-RAS*^G12V^ causes mitochondrial dysfunction [96]. Structural mitochondrial changes such as swollen, pale matrix, disorganised cristae, disruption of the mitochondrial network and aberrant shapes were observed after induction of *K-RAS*^G12V^ expression. [96]. These cells notably reduced mitochondrial respiratory chain activity [97]. More specifically, complex I was the site of the respiratory defect in the cells expressing *K-RAS*^G12V^, whereas the function of the other mitochondrial respiratory chain complexes appeared normal or slightly upregulated. Immunoblot analysis revealed that the level of the nuclear-encoded proteins, NDUFA 6,7 (20-kD component of complex I) was significantly reduced indicating complex I impairment in *K-RAS*^G12V^-expressing cells [97]. Because the mitochondrial respiratory chain produces ROS [98,99], and *K-RAS*^G12V^ causes changes in the respiratory complex, changes in ROS generation were investigated. Data showed that activating *KRAS*^G12V^ caused a rapid increase in ROS generation with lower protein levels of two major antioxidant enzymes, superoxide dismutase 2 (SOD2) and catalase. 

After prolonged *K-RAS*^G12V^ expression, the cells initiated a metabolic adaptation to mitochondrial dysfunction by increasing glycolytic activity. The connection between early mitochondrial dysfunction and the relatively late increase in glycolytic activity suggests that glycolysis upregulation was most likely a cellular response to metabolic stress caused by a decrease in mitochondrial respiration caused by *K-RAS*^G12V^ [96].

Even though mutant KRASG12D activity increases glucose uptake and reroutes glucose metabolism into the hexosamine biosynthesis and pentose phosphate pathways [100], the metabolic impact of *KRAS* mut copy gain is unknown. The glycolytic gene expression profiles of *KRAS*^G12D/WT^ and *KRAS*^WT/WT^ MEFs were similar except for *Slc2a1* (coding GLUT1) and *Slc2a3* (coding GLUT3). In contrast, glycolytic gene expression was significantly upregulated in *KRAS*^G12D/G12D^ MEFs, in agreement with increased glucose uptake, lactate secretion, and glycolytic capacity. As a result, *KRAS*^G12D^ copy gain induces a glycolytic switch, whereas a heterozygous *KRAS* mutation is insufficient to upregulate glycolysis [101].

Despite having comparable proliferative rates, cell volume, diameter, protein, and RNA content, *KRAS*^G12D/G12D^ MEFs exhibited a glycolytic switch compared to *KRAS*^G12D/WT^ cells. Furthermore, despite a *KRAS*^G12D^-associated decrease in mitochondrial membrane potential observed under homozygosity, mitochondrial morphology and function were consistent across genotypes [96,97]. Therefore, the glycolytic switch may reflect alternative glucose utilisation by *KRAS*^G12D/G12D^ cells [101]. Metabolomics analysis confirmed *KRAS*^G12D/G12D^ cells enhanced glycolytic phenotype and, unexpectedly, revealed a significant increase in glucose-derived TCA cycle metabolites in *KRAS* mut homozygous MEFs, confirming their intact mitochondrial function. More importantly, these findings revealed a *KRAS* mutant copy gain-specific metabolic rewiring as well as a glucose metabolism signature [101]. Despite their differences in glucose utilisation, *KRAS*^G12D/WT^ and *KRAS*^G12D/G12D^ MEFs had similar levels of OXPHOS, implying other TCA cycle differences. Because *KRAS* mut cells preferentially use glutamine over glucose to fuel the TCA cycle [102,103], glutamine metabolism was investigated. Glutamine-derived TCA cycle metabolites and glutamine-derived oxygen consumption increased in *KRAS*^G12D/WT^ cells, but not *KRAS*^G12D/G12D^ MEFs [101].

**Figure 3:**
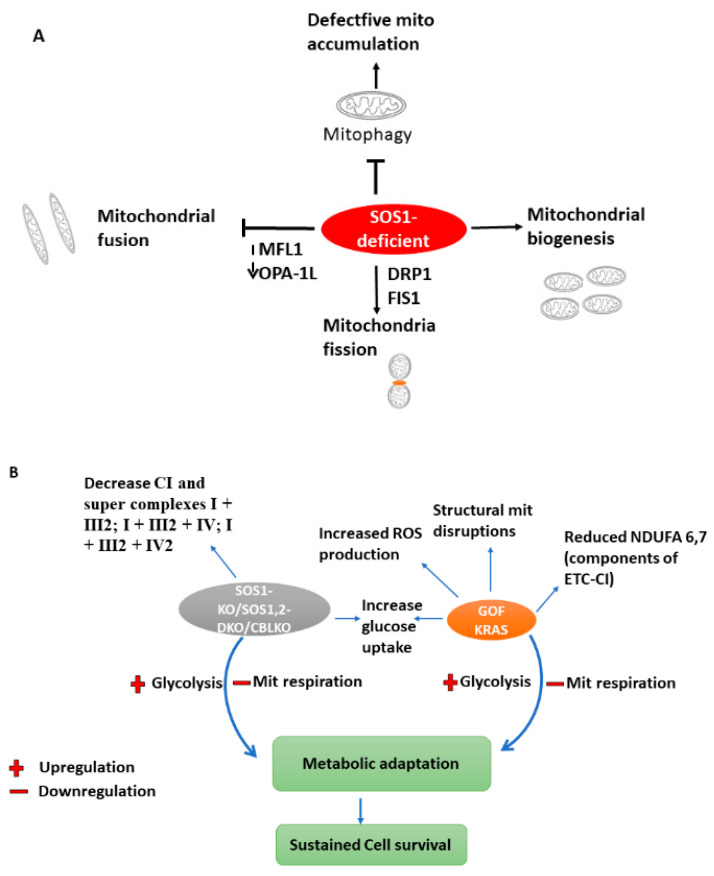
(**A**)***SOS1* alters mitochondria morphology, mass, and dynamics**. *SOS1* deficiency blocks mitophagy, hence causes accumulation of defective mitochondria. Reduced (dotted down arrow) fusion-promoting proteins such as Mitofusin 1 (MFN1) and the long-form of mitochondrial dynamin-like GTPase (OPA-1L) leads to inhibition of mitochondrial fusion while the presence of GTPase Dynamin-Related protein 1 (DRP1) and Mitochondrial fission 1 (FIS1) favours mitochondria fission, hence the changes in mitochondria morphology, mass and dynamics. (**B**)***SOS1/2* and *KRAS* mutation redirects cellular metabolism.** The actions of *SOS1*, *SOS1/2-DKO*, *CBL*-KO and GOF *KRAS* rewires cellular metabolism as a consequence of decrease in respiration chain complexes, change in mitochondria structure, and utilisation of less oxidative substrates (blue arrows).

Enhanced pyruvate dehydrogenase (Pdh) activity [104] could explain the reprogramming of homozygous cells’ glucose metabolism. However, Pdh E1 component subunit alpha (Pdhe1a) expression and Pdh activity were comparable in *KRAS*^G12D/G12D^ and *KRAS*^G12D/WT^ MEFs [101]. The authors then hypothesised that genotype-specific metabolic requirements instead drive this metabolic switch. Surprisingly, glutathione (GSH) and its precursors were significantly enriched in glucose-derived carbons in *KRAS*mut homozygous cells, implying that the *KRAS*mut copy gain rewired glucose metabolism towards glutathione biosynthesis, with glutamine even more efficiently metabolised towards GSH biosynthesis in *KRAS*^G12D/G12D^ cells MEFs. Accordingly, *KRAS*^G12D/WT^ MEFs had lower ROS levels and a higher NADPH/NADP+ ratio than *KRAS*^WT/WT^, consistently with previous findings [103,105]. Nonetheless, *KRAS*^G12D/G12D^ MEFs had a distinct antioxidant signature, as evidenced by significantly increased NADPH and GSH synthesis, NADPH/NADP+, and GSH/GSSG ratios, as well as lower ROS levels and resistance to ROS-inducing agents such as H_2_O_2_ [101]. Because the metabolic heterogeneity of *KRAS*mut cells may limit the efficacy of generalised targeting approaches [106], potential *KRAS*mut copy number-dependent susceptibilities were investigated. *KRAS*^G12D/G12D^ MEFs, unlike heterozygotes, were extremely sensitive to low glucose and to the hexokinase 2 (HK2) inhibitor 2-Deoxy-D-glucose (2DG), which triggered a considerable apoptotic response. Meanwhile, *KRAS*^G12D/WT^ MEFs were more sensitive to low glutamine levels. *KRAS*^G12D/G12D^ cells (but not *KRAS*^WT/WT^ or *KRAS*^G12D/WT^) showed high ROS levels after treatment with 2DG, confirming a reliance on glucose for efficient ROS management [101]. In summary, a prolong effect of RAS mutations on primary cells could initiate a metabolic adaptation to mitochondrial dysfunction via glycolysis upregulation accompanied by decrease mitochondria respiration (Figure 3B).

### 5.3. SOS1/SOS2 (Sons of the Sevenless)

SOS1 and SOS2, the two highly homologous mammalian orthologues of *SOS*, are the most abundant RAS-GEFs (guanine nucleotide exchange factors) in metazoan cells [107,108,109] which induce GTP/GDP exchange on cellular RAS proteins in the context of a multitude of distinct cellular surface Receptor tyrosine Kinase signalling pathways (RTKs) [107,110]. Several genetic alterations or mutations in different *SOS1* (rarely *SOS2*) domains have been identified in inherited RASopathies such as NS [14,111]. Despite the homology, data on *SOS1/2*-KO mice have shown that SOS1 is more functional than SOS2 in cellular processes such as proliferation, migration, inflammation, and maintenance of intracellular redox homeostasis; however, certain functional redundancy cannot be ruled out, especially at the organismal level [112,113].

García-Navas et al. recently investigated the relationship between SOS and mitochondrial function using primary MEFs derived from four SOS genotypes, wild-type (WT), *SOS1* single Knockout (*SOS1*-KO), *SOS2* single knockout (*SOS2*-KO) and *SOS1/2* double knockout (*SOS1/2* DKO) mice [114]. The authors evaluated various structural mitochondrial subtypes in MEFS from all four SOS genotypes [115], revealing a distinct increase in spherical-shaped mitochondrial subtypes and a decrease in tube-shaped mitochondrial subtypes in MEFs lacking *SOS1* and *SOS1/2* compared to WT and *SOS2* deficient cells [116]. Quantification of signals from mitochondrial immunofluorescence [115] showed apparent increase in the number of individual mitochondrial structures in the cytoplasm of MEFs; however, their average size was consistently smaller in *SOS1*-deficient MEFs compared to WT and *SOS2*-KO MEFs. *SOS1* and *SOS1/2*-DKO -deficient MEFs had a significant increase in mitochondrial mass compared to WT or *SOS*-KO cells [116]. The average mitochondrial morphology results from a dynamic equilibrium between fusion and fission [117]. In *SOS1*-deficient MEFs, analysis of mitochondrial fusion and fission regulators [117,118,119] revealed reduction of fusion-promoting proteins such as Mitofusin 1 (MFN1) [120] and the long-form of mitochondrial dynamin-like GTPase (OPA-1L) [121] as well as stable levels of fission promoters such as GTPase Dynamin-Related protein 1 (DRP1) and Mitochondrial fission 1 (FIS1) [122]. DRP1 expression was also higher in pure mitochondrial preparations. These findings point to a shift in the equilibrium toward mitochondrial fission in *SOS1*-deficient MEFs, implying that *SOS1* deletion results in particular changes in mitochondrial morphology, mass, and dynamics [116].

Moreover, *SOS1*-defective cells showed an increased population of defective mitochondria, and a significantly lower rate of superoxide production in *SOS1*-KO and *SOS1/2*-DKO mitochondria, confirming a severe loss of mitochondrial functionality connected to *SOS1* absence in these cell models [116,123,124].

The expression of representative OXPHOS respiratory complex subunits [125] in MEFs of the SOS genotypes was next assessed via western blotting and revealed a specific increased NDUSF3 subunit of complex I [126] and UQCRC2 subunit of complex III [127] in mitochondria lacking SOS1, particularly *SOS1/2*-DKO [116]. *SOS1*-KO and *SOS1/2*-DKO samples exhibited significantly lower formation of super complexes, including complex I (CI and super complexes I + III2; I + III2 + IV; I + III2 + IV2) as compared to the control and *SOS2* deleted genotype. Furthermore, *SOS1*-deficient samples showed a distinct form of a complex III2 + IV released from CI that was not seen in WT or *SOS2*-KO samples and much lower amounts of free complex IV [116,128]. These findings imply that SOS1 is required for the correct assembly of mitochondrial supercomplexes and that *SOS1* deficiency is associated with specific alterations in mitochondrial respiratory complex components and supercomplex assembly.

The respiratory and metabolic profiles [129] of *SOS1/2*-deficient MEFs revealed a much lower basal respiration and spare respiratory capacity compared to controls and single *SOS1* deficient cells, confirming prior findings [113]. Moreover, the loss of *SOS1* in MEFs resulted in significantly lower rates of ATP synthesis from glycolysis (glycoATP) and oxidative phosphorylation (mitoATP). MitoATP and glycoATP synthesis distinctly distinguished WT and *SOS2* from the other phenotypes, which showed much less energetic and more quiescent phenotypic profiles [116,130]. In summary, these data demonstrated that mitochondrial respiratory and metabolic defects are linked to *SOS1* ablation.

Because different cell types use different nutrient substrates to support oxidative energy metabolism [131], the Garca-Navas team evaluated whether the lack of *SOS1* in MEFs would affect the type of preferred substrates or their oxidation mechanisms. All *SOS*-deficient genotypes examined, revealed meagre glutamine oxidation rates, with the rates in *SOS1*-deficient MEFs even lower or nearly negligible when compared to controls and *SOS2*-KO cells. According to the authors, *SOS* deficient phenotypes showed different patterns of oxidation rates in the presence of endogenous or externally administered fatty acid [116].

Finally, the OCR tracings for glucose consumption showed that glucose was the preferred oxidation substrate for MEFs, and also revealed that *SOS1*-deficient cells utilize glucose as the only oxidative substrate, but they did so much less efficiently (50%) than WT or *SOS2*-KO counterparts. *SOS1/2*-DKO MEFs showed cleaved caspase-3 (CC3) in the absence of glucose, implying that the GEF activity provided by either SOS isoform is required to avoid cell death and sustain MEF survival following glucose deprivation. Addition of glucose increased hexokinase-I HK1 (but not HK2) [132] and decreased phospho-AMP-activated protein kinase (pAMPK) [133] and lactate dehydrogenase A (LDHA) in *SOS1* and *SOS1/2* deficient phenotypes when compared to the other genotypes [116]. Together, *SOS1*-deficient cells downregulate pAMPK thereby evoking specific alterations in substrate oxidation accompanied by alterations in mitochondrial respiration to sustain survival. SOS1 deficiency blocks mitophagy, hence causing accumulation of defective mitochondria. In addition, changes within the mitochondria include: reduction in fusion-promoting proteins such as Mitofusin 1 (MFN1) and the mitochondrial dynamin-like GTPase (OPA-1L) resulting to inhibition of mitochondrial fusion. Meanwhile the presence of GTPase Dynamin-Related protein 1 (DRP1) and Mitochondrial fission 1 (FIS1) favours mitochondria fission, hence the changes in mitochondria morphology, mass and dynamics summarises the mechanistic insight of *SOS* mutation on energy metabolism. (Figure 3A,B).

### 5.4. CBL

Casitas B-lineage Lymphoma (*CBL*) is a proto-oncogene found mutated in NS patients [25,134,135]. *CBL* encodes an E3-ubiquitin ligase from the *CBL* family that mediates protein ubiquitination and, once heterozygously mutated, causes abnormalities clinically similar to NS patients [25,26,136]. This protein has a phosphotyrosine-binding domain at its N-terminus that allows it to engage with a variety of tyrosine-phosphorylated substrates and target them for proteasomal or enzymatic destruction. As a result, CBL proteins act as a negative regulator in various signal transduction pathways, including P13K/AKT pathway [137].

Previously, studies performed on *CBL*-knockout and knockdown monocyte cells and mice revealed significant enlargement in mitochondria size and high energy expenditure [137,138]. *CBL*-KO and wild-type human monocytic cell line (THP-1)-derived macrophages had higher glycolytic capacity, glycolytic reserve rates and elevated OXPHOS than WT cells [139]. Since glycolysis is required for NLRP3 inflammasome activation, Lin et al. further investigated the mechanisms involved in *Cbl*-mediated glycolytic control [139]. In a previous study, *Cbl*-deficient mice showed a noticeable increase in glucose tolerance [138], suggesting that *Cbl* inhibition may enhance the transportation of glucose inside the cells. The uptake of the glucose analogue 2-NBDG was significantly higher in *CBL*-KO cells or in WT cells treated with the *Cbl* inhibitor hydrocotarnine compared with the untreated control. Thus, these findings imply that *Cbl* negatively contrlos cellular glucose uptake in THP-1-derived macrophages [139].

Cellular glucose uptake occurs through glucose transporters (GLUTs) [140,141,142,143], with GLUT1 being the most abundant in macrophages [144]. In fact, the surface expression of GLUT1 protein in *CBL* deficient cells was higher than in WT cells [139]. In summary, Lin’s research demonstrated that inhibiting *Cbl* enhanced cellular glucose uptake, glycolytic capability, and mitochondrial OXPHOS capacity [139].

Together, these data suggest that *CBL* mutations not only affect mitochondria size and energy expenditure but also mitochondrial respiration via glucose upregulation.

### 5.5. RRAS

Because of the large number of genes mutated in NS, approximately 10–20% of diagnosed patients do not have mutations in previously known NS genes, implying that additional unidentified genes may be present and contribute to the disease onset. In a recent study, Flex et al. described a new disease-associated gene called *RRAS* carrying a GOF mutation and encoding a small monomeric GTPase that controls cell adhesion, spreading, and migration, underlying a rare and variable phenotype with features that partially overlap NS [31]. Later, Capri et al. also identified activating *RRAS2* mutation in six unrelated subjects/families with NS [30].

Despite identifying this gene in NS patients, there is still little known about the effects of hyperactivated *RRAS* on mitochondria and energy metabolism. Alcover and coworkers recently studied *RRAS2* mutation in hypomyelinated disease-mouse models. The authors hypothesised that the cause of hypomyelination in vertebrates could be associated with metabolic alterations since axonal synaptic transmission depends on myelin insulation and metabolic support. Understanding the mitochondria adaptations in knockout RRAS2 phenotype gave insights into the degree of axonal myelination. In *R-Ras2*/(*R-Ras2*KO) mice, where fewer axons were myelinated, the number of mitochondria in nonmyelinated axons increased, suggesting dysfunction in these mitochondria structures [145]. However, the defects or alterations associated with mitochondrial dysfunction in nonmyelinated axons are still unknown. According to the authors, the physiological outcome of *RRAS* loss-of-function in human disorders could promote axonal degeneration and hypomyelination [145]. 

### 5.6. RAF 

Among the RAF protein family isoforms (B-RAF, C-RAF) encoded by the *RAF* gene and found mutated in NS patients, *C-RAF* (also called *RAF1*) is the only one with a direct target on the mitochondria [146]. The amino-terminal domain of active C-RAF binds the mitochondrial surface with high affinity and regulates mitochondrial shape and cellular distribution through its coupling with MEK [146] though the mechanism remains unclear. Such morphologic alterations of the mitochondrial network are involved in cell migration and Ca^2+^ metabolism in endothelial cells [147,148]. Wang discovered that proteins encoded by the active *C-RAF* gene regulate mitochondrial function by phosphorylating outer mitochondrial membrane proteins such as BAD (a pro-apoptotic BCl-2 homolog), subsequently phosphorylating ERK-1 and 2 thus inhibiting apoptosis [149]. A recent study of patients clinically diagnosed with NS and NSML revealed the presence of *RAF1* and *BRAF* pathogenic variants, with these patients also displaying systemic disorders such as congenital heart disease [35,150]. C-RAF is an essential effector of Ras-mediated signalling and a critical regulator of the ERK/MAPK pathway [151]. The cooperation of C-RAF with intracellular kinases such as PAK5 (a serine/threonine-protein kinase that targets C-RAF to mitochondria in a serine 338 independent manner) regulates C-RAF activity and controls the C-RAF-dependent Raf/MEK signalling at the mitochondria [152]. An Inhibitory kinase of C-RAF, termed RAF kinase inhibitory protein (RKIP), block its translocation to the mitochondrial membrane [153]. However, fibroblast growth factor (FGF) induces C-RAF mitochondrial translocation in vascular endothelial cells by phosphorylating its Ser338 (S338) [154]. Overexpression of specific intracellular proteins such as Bcl-2 (regulator of apoptosis) recruits C-RAF into the mitochondria conferring cell survival upon dimerisation and phosphorylation of the stress-activated pro-apoptotic kinase MST2 [155]. Furthermore, C-RAF and B-RAF, independent of the usual RAF signalling pathway, can induce some metabolic features (such as fusion, fission, biogenesis and mitophagy) mediated by mitochondria [155,156]. The distinct role of C-RAF in the mitochondria provides compelling evidence for targeting mitochondrial RAF proteins to maintain mitochondrial homeostasis [149,157].

Tsai et al., examined mitochondria function and ROS production of wild-type and C-RAF knockout MEF treated with RAF inhibitors sorafenib and GW5074. There was a 6-fold increase in ROS production and a decrease in mitochondrial membrane potential in *C-RAF*^-WT^ but not in *C-RAF^-^*^/-^. Furthermore, the therapy induced mitochondria fragmentation with donut and blob-shaped formation in *C-RAF*^-WT^ but not *C-RAF^-^*^/-^ MEFs. Thus, these findings suggest that inhibiting *C-RAF* causes mitochondrial dysfunction, which may have contributed to cell death [158]. Moreover, C-RAF interacts with Ser308 of phosphorylated DAPK (Death-associated protein kinase), directing its colocalisation to the mitochondria for regulating mitochondrial modelling [158].

**Figure 4:**
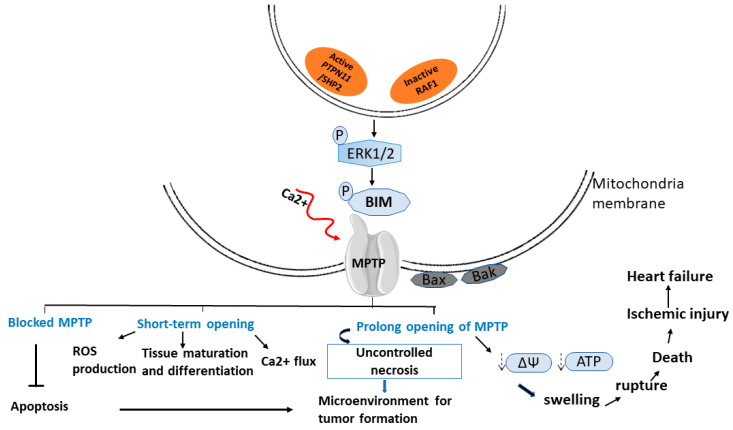
**Effect of *PTPN11* and *RAF1* mutation on mitochondria permeability transition pore (mPTP).** Upon *PTPN11* activation or *RAF1* inactivation, ERK signalling is phosphorylated, in turn phosphorylating and activating apoptotic regulators such as Bcl-2-like protein 11 (BIM); Bcl-2-like protein 4 (Bax), and Bcl-2 homologous antagonist/killer (Bak) localized on the mitochondrial membrane. This event destabilizes the opening and closure of the mitochondria permeability transition pores (mPTP), causing a blocked or prolonged opening state of the membrane channels. Thus, a huge efflux of Ca2+ (prolong state) is observed, resulting in decreased mitochondrial membrane potential and altered and uncontrolled cellular processes.

The inhibition of *RAF1* expression resulted in low conductance gating of the mitochondrial permeability transition pore (mPTP) (Figure 4), with a decrease in the inner mitochondrial membrane potential in rats or cells lines treated with MEDICA (MEthyl-substituted DICarboxylic Acids) analogues, known inhibitors of *RAF1* [159]. Previously, MEDICA analogues were demonstrated to suppress basal P-Raf1 (Ser-338), indicating inhibition of Raf1 kinase activity [160]. In this light, the inhibition of RAF1 by MEDICA analogues could be attributed to the suppression of RAF1 expression and its kinase activity. The role of *RAF1* as an upstream target for MEDICA analogues was confirmed further by measuring cAMP levels and P-Bad (Ser-155) in COS-1 cells that overexpress *RAF1*. RAF1 overexpression prevented the decrease in cAMP and P-Bad (Ser-155) (Figure 4), indicating that RAF1 suppression may account for low conductance-MPTP gating by MEDICA analogues. Over the years, low conductance gating has been characterised by the continuous passage/flow of ions across the mitochondria membrane [161]. Thus, Samovski’s group suggested that this low conductance gating following *RAF1* mutation might have resulted from the prolonged opening of mPTP [159].

**Figure 5:**
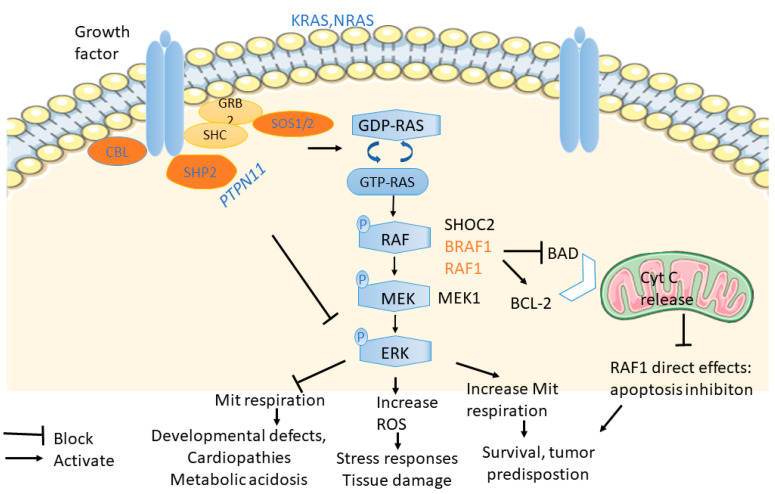
**An overview of the effects of NS/NSML mutations on mitochondria metabolism via RAS signalling.** The sequence of events coordinated by the major RAS/MAPK signalling and other pathways such as P13K/AKT results from the activation or inactivation of heterozygous/homozygous mutations in genes: *PTPN11, SOS1/2, RAS, RAF1, BRAF1, NRAS, KRAS, CBL* that cause NS and related syndrome. Outcomes observed include decreasing or increasing mitochondria metabolism, increasing ROS, and inhibiting apoptosis.

The role of these mutations in Ras signaling and their impact on mitochondrial metabolism are summarised in Figure 5. 

## 6. Conclusions

*PTPN11* accounts for the majority of germline mutations responsible for NS and NSML, followed by *SOS1, KRAS* isoform (except *HRAS*), *RAF1* and *SHOC2*. These genes play an essential role in the pathogenesis of mitochondrial/metabolic-related disorders. Exploring the bioenergetics of Noonan syndrome in mouse models and human cell lines is required to fully understand the effect of *RAS* hyperactivation on mitochondrial function and tissue development processes. This analysis will provide insights into energy metabolism and the onset of various clinical complications. 

In most cases, research has revealed that the whole action of any one of the germline mutations requires interaction and cooperation with intracellular proteins, kinases, transporters, or transcription factors (such as GLUT1, Gas6, STAT3, GEF, ROCK2, cAMP, p-AMPK, p- Bad) involved in the MAPK/ERK signalling. This has been seen most prominently in *RAF*, *KRAS*, *PTPN11*, and *SOS1* mutations [80,81,82,85,101,116,139,159,162].

Findings have shown that different mutations (homozygous or heterozygous, GOF or non-functional) on the same gene could cause heterogeneous pathological effects, which may depend on the different impacts on the mitochondria and energy metabolism [69,70]. Furthermore, not only has a single gene mutation been linked to a defect in energy metabolism but copy variants and double mutations have also been identified as having functional implications [101,116].

The longer time an active *RAS* (*KRAS*) mutant is expressed on a cell, it leaves the cell with persistent dysfunctional mitochondria resulting in a metabolic adaptation [96]. A profound influence of GOF *PTPN11* (SHP2) mutation on mitochondrial cytochrome oxidase and cytochrome C led to a decrease in mitochondrial membrane permeability (MMP), increased ROS production, and decreased ATP production [7]. These changes signal bioenergetic stress and may result in the release of apoptotic factors, culminating in cell death and accumulated ROS, which may cause oxidative damage in tissues, potentiating tissue failure [84,101,163,164]. Interestingly, it was also found that SHP2 alone is not responsible for the changes in energy metabolism, but it does require hormonal support to mediate specific cellular processes, including glucose homeostasis [70]. As a result, targeting hormonal pathways in SHP2 mutations may be critical for regulating energy metabolism. 

According to other observations, the major causes of alterations in respiration and ATP production rates during OXPHOS or changes in glycolytic rate can be attributed to mitochondrial defects, i.e., changes in mitochondrial shape, mass, and dynamics, lower or extreme high activity rates of the ETC, changes in respiratory complexes and their assembly into mitochondrial supercomplexes, abnormal levels of mitochondrial proteins (VDAC, TOM20, and TIM13), and dysfunctional MPTP, as well as the distinct patterns of substrate preference for oxidative energy metabolism and cells’ reliance on glucose for survival [113,116,159,165].

Although cognitive impairment has been reported in NS/NSML patients [166,167], there is still a lack of evidence on how energy metabolism is affected in this condition, particularly when looking at the most frequently mutated genes in NS individuals. 

Because heterozygous expression of an NS/NSML (LEOPARD syndrome) mutant of SHP2 enhances energy metabolism and protects against obesity and diabetes [69], one might wonder if equivalent pharmacological modulation of SHP2, that functionally mimics NSML mutations, could be a powerful strategy to alleviate obesity and associated pathologies. Indeed, it has been shown that SHP2 expression is upregulated in metabolic tissues of obese animals, suggesting that increased SHP2 expression/activity contributes to the development of obesity [69,70,85].

NS/NSML mutations alter metabolism via enhanced phosphorylation or decreased phosphorylation of target proteins in the MAPK/ERK pathway [70,82,85]. In addition, RAS mutation in primary human cells revealed inhibited mitophagy by activating LC3B (autophagy-related protein) in a Jun N-terminal kinase (JNK) dependent manner. Because mitophagy is only one of the mechanisms that sheds light on mitochondrial impairment in cells, inhibiting mitophagy through altered RAS allowed cells to accumulate defective mitochondria, resulting in decreased mitochondrial respiration due to insufficient glucose uptake by the cells [168]. The molecular mechanism linking RAS activation and mitophagy inhibition in primary cells should be investigated further. Whether the observed effects on mitochondrial or metabolic functions are secondary events due to main changes in other relevant processes linked to Ras signalling, this cannot be ruled out. To support the findings of Kim et al. [80] a similar study involving *Gas6* and active *Ptpn11* mutation should be carried out but in this case, assessing the mitochondrial respiratory function. Some authors have demonstrated mechanistic insights into the indirect regulation of other processes controlling mitochondrial homeostasis by altered Ras signalling [169], In such study, *HRAS* was shown to cooperate by inhibiting several modulators linked to AMPK to alter mitochondrial function in Rasopathies [169].

Although several genes are mutated in NS children, i.e., *NRAS*, *RIT1*, *A2ML1*, *SHOC2*, *RASA2*, and *LZTR1* experimental evidence of their action on mitochondrial function is not available to date. Further investigations are needed to understand the mechanisms linking NS to mitochondrial dysfunction and altered energy metabolism. In addition, we still lack insights into the outcomes of *Cbl* and *SOS1/2* activation on mitochondria metabolism since the available data are limited to the effects of *Cbl* and *SOS1/2* deletion or deficiency. Determining the role of *Cbl* and *SOS1/SOS2* GOF mutation on mitochondrial function is therefore critical. Because of the location of SHP2 on the mitochondria and its numerous disruptive effects, as discussed here, future research should focus on identifying potent pharmacological regulators that compete with SHP2 ligands/substrates, as this may be critical for alleviating complications in NS/NSML patients. Furthermore, the discussed mechanisms and pathways may serve as future targets for therapeutic solutions.

To sum up, the knowledge of mitochondrial dysfunction in syndromic, Ras/MAPK pathway-related gene mutation carriers is beneficial for accurate diagnosis and applying the appropriate treatment to a pathological condition. Research into the underlying signalling pathways and mutations in NS/NSML children that cause mitochondrial dysfunction and abnormal energy metabolism is critical for future prognosis assessment, genetic counselling, and plausible therapy.

## Figures and Tables

**Table 1:** Germline mutations of RAS/MAPK pathway responsible for NS and NSML phenotype.

Disease	Gene	Location	Amino Acid Change	Mutation Rate (%)	Protein Name	Protein Class	REF
NS	*PTPN11*	12q24	p.D61G, p.G60A	50–60	SHP2: Protein tyrosine phosphatase non-receptor type 11; Src Homology 2	Phosphatase	[7,19]
	*SOS1*	2p22-p21	p.A1654G	10–13	Son of sevenless (SOS) homolog 1	RasGEF	[20]
	*RAF1*	3p25.2	p.R89L	5–10	v-Raf-1 murine leukemia viral oncogene homolog 1	Kinase	[20]
	*KRAS*	12p12.1	p.G12X (X = any amino acid) c.A458T p.D153V p.Y71D	10	V-Ki-Ras2 Kirsten rat sarcoma viral oncogene homolog	GTPase	[20,21,22]
	*NRAS*	1p13.2	p. G12X p.Q61X	<1	Neuroblastoma RAS viral (v-RAS) oncogene homolog	GTPase	[20,23,24]
	*SHOC2*	10q25.2	p.S2G	12	soc-2 suppressor of clear homolog	Scaffolding	[20]
	*CBL*	11q23.3	p.Y371X p.C404R p.W408R p.G415V p.L380P p.C840W	<1	Casitas B-lineage lymphoma	Ubiquitin Ligase	[20,25,26]
	*BRAF*	7q34	p.V600E	<1	Serine/Threonine-Protein Kinase B-Raf	Kinase	[24]
	*A2ML1*	12p13.31	p.R802H, p.R592L, p.R802L	<1	α-2-macroglobulin-like 1	Protease inhibitor	[27]
	*SOS 2*	2p22.1	p.T376SL	<1	Son of sevenless homolog 2	RasGEF	[28]
	*LZTR1*	22q11.21	p.R284C, p.H287Y, p.Y119C, p.G248R, p.S247 N	<1	Leucine Zipper-Like Transcription Regulator 1	Adaptor protein	[20,28]
	*RASA2*	3q23	p.Y326C, p.Y326N, p.R511C	<1	Ras P21 Protein Activator 2	RasGAP	[29]
	*RRAS*	19q13.33	p.G39dup, p.V55M	<1	Related RAS Viral (R-Ras) Oncogene Homolog	GTPase	[30,31]
	*RIT1*	1q22	p.S35 T, p.A57G, p.E81G, p.F82V, p.F82L, p.T83P, p.Y89H, p.M90I, p.G95A	7	10 Ras-Like Without CAAX 1	GTPase	[20,32]
NSML	*PTPN11*	12q24	p.Q510P	90	SHP2: Protein tyrosine phosphatase non-receptor type 11; Src Homology 2	Phosphatase	[20,33,34]
	*RAF1*	3p25.2	p.V263G p.S257 L	5	v-Raf-1 murine leukemia viral oncogene homolog 1	Kinase	[24,35]
	*BRAF*	7q34		<1	Serine/Threonine-Protein Kinase B-Raf	Kinase	[24]

## Data Availability

Not applicable.

## Abbreviations

2DG	2-Deoxy-D-glucose
2-NBDG	2-(N-(7-Nitrobenz-2-oxa-1,3-diazol-4-yl)Amino)-2-Deoxyglucose
3T3-L1	3-day transfer, inoculum 3 x 105 cells- fibroblast-like cell line
8GM-CSF	Granulocyte-macrophage colony-stimulating factor
ADP	Adenosine diphosphate
AMP	Adenosine monophosphate
AMPK	AMP-activated protein kinase
ATGL	Adipose triglyceride lipase
ATP	Adenosine triphosphate
BAD	BCL2 associated agonist of cell death
BAF3	IL-3 dependent murine pro B cell
Bak	Bcl-2 homologous antagonist/killer
Bax	Bcl-2-like protein 4
BCL-2	B-cell lymphoma 2
Beta-MHC	Beta-Myosin heavy chain
BIM	Bcl-2-like protein 11
CAAX	C-Cysteine, AA-2 Aliphatic residues, X-any C-terminal amino acid
CBL-KO THP	Human CBL knockout HEK-293T cell line
CC3	Cleaved caspase-3
CcO	Cytochrome C oxidase
CI,II,III,IV	Complex I-IV
CMPs	common myeloid progenitors
COII	Cytochrome C oxidase subunit 2
COS-1	kidney fibroblast-like cell line /CV-1 (simian) in Origin, and carrying the SV40 genetic mate
Cyt c	Cytochrome c
DAPK	Death-Associated Protein Kinase
DRP1	Dynamin-related protein 1
ECAR	Extracellular acidification rate
EGF	Epidermal growth factor
EGFR	Epidermal growth factor receptor
EPO	Erythropoietin
ER	Endoplamic reticulum
ETC	Electron transport chain
FADH2	flavin adenine dinucleotide
FCCP	Carbonyl cyanide-p-trifluoromethoxyphenylhydrazone
FGF	Fibroblast growth factor
FIS1	Mitochondrial fission 1 protein
FRS-2	FGF receptor substrate 2
Fru1,6bisP	Fructose-1-bisPhosphate
Gab1	GRB2-associated-binding protein 1
GDP	Guanosine diphosphate
GF	Growth factor
GLUT1	Glucose transporter 1
GLUT3	Glucose transporter 3
glycoATP	Glycolytic ATP
GM-CSF	Granulocyte macrophage-colony stimulating factor
GMPs	Granulocyte macrophage progenitors
GOF	Gain-of-function
GRB2	Growth factor receptor-bound protein 2
GSH	Glutathione
GSH/GSSG	Glutathione/Glutathione disulfide (reduced glutathione/Oxidised glutathione) ratio
GTP	Guanosine-5′-triphosphate
GTPase	GTP hydrolase
GW5074	3-(3,5-Dibromo-4-hydroxybenzyliden)-5-iodo-1,3-dihydroindol-2-one (a C-RAF inhibit)
HEK293	Human embryonic kidney 293 cells
HFD	Hight fat diet
HGP	hepatic glucose production
HK1/HK2	Hexokinase 1 and 2
HSCs	Hematopoietic stem cells
HSL	Hormone-sensitive lipase
IL-3	Interleukin 3
IMS	intermembranous space
IRS-1	Insulin receptor substrate 1
Jak2	Janus kinase 2
JMML	Juvenile myelomonocytic leukemia
JNK	c-Jun N-terminal kinases
KO	Knock-out
LC3B	Light chain 3B (subunit of Microtubule-associated proteins 1A/1B)
LDHA	Lactate Dehydrogenase A
LIF	Leukemia inhibitory factor
LSHKO	Liver-specific Shp2 Knock-out
MAPK/ERK	Mitogen-activated protein kinase/Extracellular signal-regulated kinase 1/2
MDC	Mitochondria disease criteria
MEDICA	MEthyl-substituted DICarboxylic Acids
MEF	Mouse embryonic fibroblast
MEK1 or MAP2K1	Dual specificity mitogen-activated protein kinase kinase 1
MEPs	Megakaryocyte erythroid progenitors
MFN1	Mitofusin-1 protein
Mit	Mitochondria
mitoATP/mitATP	mitochondria ATP
MitoSOXTM	Red Mitochondrial Superoxide Indicator
MitoTrackerTM Red CMXRos	MitoTracker Red and Chloromethyl-X-rosamine
MMP	Mitochondrial membrane potential
mPTP	Mitochondrial permeability transition pores
MST2	mammalian Sterile 20-like kinase 2
mtDNA	Mitochondrial DNA
mTOR/S6K	mammalian target of rapamycin (mTOR)/ribosomal protein S6 kinase
mut	mutation
MYBPC3	Cardiac myosin binding protein C3
NADH	Nicotinamide adenine dinucleotide
NADP+	Oxidised form of Nicotinamide adenine dinucleotide phosphate (NADPH)
ND	NADH Dehydrogenase
NDUSF3	NADH dehydrogenase [ubiquinone] iron-sulfur protein 3
NLRP3	NLR family pyrin domain containing 3
NSC87877	8-Hydroxy-7-[(6-sulfo-2-naphthyl)azo]-5-quinolinesulfonic acid (Inhibitor of SHP2/SHP1)
OCR	Oxygen consumption rate
OGTT	Oral glucose tolerance test
OPA-1L	Dominant optic atrophy (long form of mitochondrial dynamin-like GTPase)
OXPHOS	Oxidative phosphorylation
pAMPK	phospho-AMP-activated protein kinase
Pdh	Pyruvate dehydrogenase
Pdhe1a	Pyruvate dehydrogenase E1 component subunit alpha
p-ERK	Phosphorylated ERK
PI3K-AKT-mTOR	The phosphatidylinositol-3-kinase (PI3K)/Akt and the mammalian target of rapamycin (mTOR)
PP1C	Protein phosphatase 1 catalytic subunit
PPP	Pentose phosphate pathway
RAS/MAPK	Rat sarcoma virus/Mitogen-activated protein kinase
RB2	Retinoblastoma-like protein 2
ROS	Reactive oxygen species
RTK	Receptor tyrosin kinase
SOD2	Superoxide dismutase 2
SOS1/2-DKO	Double knock-out SOS1/2
SOS1-KO	Single Knock-out SOS1
SOS2-KO	Single Knock-out SOS2
SRC	Spare respiratory capacity
STAT3	Signal transducer and activator of transcription 3
TCA	Tricarboxylic acid cycle
THP-1	Tamm-Horsfall Protein 1 (Spontaneously immortalized monocyte-like cell line)
TIM13	Translocase of inner membrane 13
TOM20	Translocase of outer membrane 20
TOMM40	Translocase of outer mitochondria membrane 40
UQCRC2	Cytochrome b-c1 complex subunit 2
VDAC	Voltage-dependent anion channels (mitochondrial porins)
WT	Wildtype
